# High Fibroblast Growth Factor 23 concentrations in experimental renal failure impair calcium handling in cardiomyocytes

**DOI:** 10.14814/phy2.13591

**Published:** 2018-04-02

**Authors:** Melissa Verkaik, Maarten Oranje, Desiree Abdurrachim, Max Goebel, Zeineb Gam, Jeanine J. Prompers, Michiel Helmes, Pieter M. ter Wee, Jolanda van der Velden, Diederik W. Kuster, Marc G. Vervloet, Etto C. Eringa, the NIGRAM consortium

**Affiliations:** ^1^ Department of Nephrology VU University Medical Center Amsterdam The Netherlands; ^2^ Department of Physiology Amsterdam Cardiovascular Sciences VU University Medical Center Amsterdam The Netherlands; ^3^ Biomedical NMR Department of Biomedical Engineering Eindhoven University of Technology Eindhoven The Netherlands

**Keywords:** Calcium, cardiac, chronic kidney disease, FGF23, mineral bone disease

## Abstract

The overwhelming majority of patients with chronic kidney disease (CKD) die prematurely before reaching end‐stage renal disease, mainly due to cardiovascular causes, of which heart failure is the predominant clinical presentation. We hypothesized that CKD‐induced increases of plasma FGF23 impair cardiac diastolic and systolic function. To test this, mice were subjected to 5/6 nephrectomy (5/6Nx) or were injected with FGF23 for seven consecutive days. Six weeks after surgery, plasma FGF23 was higher in 5/6Nx mice compared to sham mice (720 ± 31 vs. 256 ± 3 pg/mL, respectively, *P* = 0.034). In cardiomyocytes isolated from both 5/6Nx and FGF23 injected animals the rise of cytosolic calcium during systole was slowed (−13% and −19%, respectively) as was the decay of cytosolic calcium during diastole (−15% and −21%, respectively) compared to controls. Furthermore, both groups had similarly decreased peak cytosolic calcium content during systole. Despite lower cytosolic calcium contents in CKD or FGF23 pretreated animals, no changes were observed in contractile parameters of cardiomyocytes between the groups. Expression of calcium handling proteins and cardiac troponin I phosphorylation were similar between groups. Blood pressure, the heart weight:tibia length ratio, *α*‐MHC/*β*‐MHC ratio and ANF mRNA expression, and systolic and diastolic function as measured by MRI did not differ between groups. In conclusion, the rapid, CKD‐induced rise in plasma FGF23 and the similar decrease in cardiomyocyte calcium transients in modeled kidney disease and following 1‐week treatment with FGF23 indicate that FGF23 partly mediates cardiomyocyte dysfunction in CKD.

## Introduction

Patients with chronic kidney disease (CKD) are at increased risk for cardiovascular diseases. Importantly, these patients are more likely to die from cardiovascular causes than reaching end‐stage renal disease (ESRD) (Sarnak et al. 2003; Go et al. 2004). Cardiovascular diseases associated with impaired kidney function include heart failure, left ventricular hypertrophy (LVH), stroke, peripheral artery disease, coronary heart disease and atrial fibrillation (Levin et al. 1996; Abramson et al. 2003; Keith et al. 2004; Foley et al. 2005; Astor et al. 2006; Kottgen et al. 2007; Wattanakit et al. 2007; Alonso et al. 2011), of which heart failure is the predominant complication among patients with CKD (Segall et al. 2014). CKD‐associated mortality is worse in diastolic heart failure patients than in those with systolic heart failure (Ahmed et al. 2007) and diastolic dysfunction is already present in early stages of CKD (Hayashi et al. 2006; Otsuka et al. 2009).

Traditional cardiovascular risk factors, such as old age, diabetes mellitus type II, hypertension, and hyperlipidemia are highly prevalent in CKD, but only partially explain the high cardiovascular mortality rate (Sarnak et al. 2003). This suggests that other factors in CKD contribute to this increased cardiovascular risk. Fibroblast growth factor 23 (FGF23) has been proposed to be such a factor (Scialla et al. 2014).

Already in early stages of CKD, patients with higher concentrations of FGF23 face an increased risk of all‐cause mortality and of progression to ESRD compared to patients with lower FGF23 concentrations (Isakova et al. 2011; Kendrick et al. 2011). In addition, in several clinical cohort studies strong associations were found in CKD patients of all stages between increased FGF23 levels and increased risk of cardiovascular disease (Seiler et al. 2010; Kendrick et al. 2011; Brandenburg et al. 2014). FGF23 has been linked directly to LVH, both in CKD and non‐CKD patients(Gutierrez et al. 2009) and experimental studies demonstrated that FGF23 can directly induce cardiomyocyte hypertrophy and disturbed calcium fluxes (Faul et al. 2011; Touchberry et al. 2013). Moreover, FGF23 has been linked to impaired LV function, even in the absence of LVH (Seiler et al. 2011), but functional data explaining how FGF23 disturbs cardiomyocyte function are lacking.

In summary, the molecular changes that may underlie the increased prevalence of heart failure and cardiac mortality in CKD are poorly understood. Based on compelling epidemiological evidence linking FGF23 to CKD‐related cardiomyopathy, we hypothesized that CKD impairs cardiac function, besides established structural change. We hypothesize this is due to a direct effect of high FGF23 concentrations on cardiomyocyte contraction and relaxation, by modifying calcium fluxes in cardiomyocytes.

## Methods

### Animals

Six‐week‐old male wild‐type C57BL/6 mice (Charles River Laboratories, Leiden, The Netherlands) were housed under standardized conditions. All mice received water and food ad libitum. All experiments were approved and conducted following the guidelines of the local animal ethical committee and complied with Dutch government guidelines.

### Induction of CKD

Seven‐week‐old male mice were randomized to either 5/6 nephrectomy (5/6Nx) as described before (Bro et al. 2003), or sham surgery under isoflurane anesthesia and preoperative analgesia (Buprenorphine; Temgesic (Schering‐Plough), 0.05 mg/kg intramuscular). In short, an abdominal dorsal midline incision of the skin and muscles was made and the left kidney was decapsulated to avoid ureter and adrenal damage, after which the upper and lower poles were removed by a bipolar electrocoagulator. In the same procedure, the complete decapsulated right kidney was removed after ligation of the renal blood vessels and the ureter. After surgery, all mice received subcutaneous injections of postoperative analgesia for two consecutive days (Ketoprofen; Ketofen (Merial S.A.S.), 5 mg/kg). In control mice, sham surgery was performed, which included decapsulation of both kidneys, but no removal of kidney tissue. The remainder of the protocol was followed as for the 5/6Nx group. Six weeks after surgery mice were placed into individual metabolic cages (Tecniplast, Milan, Italy) for collection of 24‐h urine samples. Evaporation of urine was minimized by the addition of paraffin oil to the collection tube.

### Exogenous FGF23 supplementation

13‐week‐old male mice were randomized and injected intraperitoneally twice daily for seven consecutive days with either vehicle (phosphate buffered saline, PBS) or 160 *μ*g/kg/day recombinant mouse FGF23 (8 *μ*g/mL) (de Jong et al. 2017) (6His‐tagged Tyr25‐Val251 (Arg179Gln); 26.1 kDa, R&D Systems, Minneapolis, MN, USA). After 7 days seven mice were placed into individual metabolic cages as described above.

### FGF23 measurements in plasma samples

Blood was collected by either tail vein incision or heart‐puncture at the end of all experiments. Blood was put into EDTA‐coagulated microtainers (BD Microtainer tubes, Plymouth, UK) and centrifuged for 10 min at 1500 *g* at 4°C. Plasma samples were stored at −80°C. Circulating C‐terminal FGF23 concentrations from EDTA‐ anticoagulated plasma were measured in duplicate using an ELISA assay (Immutopics International, San Clemente, CA, USA), according to the manufacturers protocol.

### Intact cardiomyocyte isolation and mechanics

Intact ventricular cardiomyocytes were isolated as described previously (King et al. 2011). Briefly, the heart was dissected placed in ice‐cold perfusion buffer (composition: 113 mmol/L NaCl, 4.7 mmol/L KCl, 0.6 mmol/L KH_2_PO_4_, 12 mmol/L NaHCO_3_, 10 mmol/L KHCO_3_, 30 mmol/L Taurine, 1.2 mmol/L MgSO_4_, 10 mmol/L HEPES, 0.6 mmol/L Na_2_HPO_4_, 5.5 mmol/L glucose, 9.9 mmol/L BDM, 12.4 *μ*mol/L CaCl_2_). The heart was then cannulated via the aorta to the Langendorff apparatus. After perfusion with perfusion buffer and digestion buffer (perfusion buffer with 0,9 mg/mL collagenase (Type II, 265 U mg^−1^; Worthington Biochemical, NJ, USA)) at 37°C and pH 7.4, the heart was placed in a myocyte stopping buffer (perfusion buffer with 50 *μ*L/mL fetal bovine serum). The aorta and atria were removed, and the ventricles were cut into small pieces. After triturating with a plastic Pasteur pipette, the cell suspension was filtered through a 250‐*μ*m nylon mesh filter and supernatant was discarded after cell gravity pelleting. Calcium was reintroduced to a final concentration of 1.0 mmol/L. Finally, cells were washed twice with Tyrode solution (1.0 mmol/L Ca^2+^, 133.5 mmol/L NaCl, 5 mmol/L KCl, 1.2 mmol/L NaH_2_PO_4_, 1.2 mmol/L MgSO_4_, 10 mmol/L HEPES, 11.1 mmol/L glucose, 5 mmol/L sodium pyruvate). Calcium was reintroduced to a final concentration of 1.8 mmol/L.

Unloaded shortening and Ca^2+^ transients of intact mouse cardiomyocytes were monitored as described before (Pohlmann et al. 2007). In short, cardiomyocytes were incubated in Tyrode solution containing 1 *μ*mol/L Fura‐2 acetoxymethyl ester (Fura‐2 AM) for 15 min and washed for a period of 12 min in Tyrode solution. Single cardiomyocytes (without spontaneous contraction) were placed into a temperature‐controlled (37°C) and randomly selected, and sarcomere shortening as well as relaxation kinetics and Ca^2+^ transient were monitored at 1 Hz electrical field stimulation (20V, 0.4 msec pulses). Sarcomere shortening and relaxation were visualized using a video‐based sarcomere length (SL) detection system (IonOptix corporation, Milton, MA, USA). To measure Ca^2+^‐transients, cardiomyocytes were excited at 340 nm and 380 nm with emission monitored at 510 nm. The F340/F380 fluorescence ratio was used as a measure of cytosolic [Ca^2+^].

### Protein and phosphorylation analysis by Western blot

To assess calcium channel changes, western blotting analysis of SERCA2a was performed using a monoclonal rabbit antibody (courtesy of Warner S. Simonides, VU University Medical Center; dilution 1:4000) and was corrected for *α*‐actin expression (Sigma‐Aldrich, A3853‐100UL; dilution 1:5000) (de Waard et al. 2009). Western blotting analysis of site‐specific phosphorylation of (phospholamban) PLN at the serine (Ser)‐16 site was performed using antibody against PLN phospho Ser‐16 (Badrilla, A010‐12; dilution 1:1000) and total PLN protein levels were determined, using an antibody against total PLN (Badrilla, A010‐14; dilution 1:1000) (Najafi et al. 2016). Cardiac TnI (Thermo fischer scientific # MA 122700; dilution 1:100,000) phosphorylation status of frozen heart tissue samples was analyzed, using one‐dimensional sodium dodecyl sulfate polyacrylamide‐bound Mn^2+^‐phos‐tag gel electrophoresis and western blotting, as described before (Sequeira et al. 2013).

### Cardiac function measurement by MRI

Cardiac systolic and diastolic function were measured by MRI using a 9.4 T horizontal bore MR scanner (Bruker, Germany), and a 35‐mm quadrature birdcage coil (Bruker, Germany) for both signal reception and transmission, as described before (Coolen et al. 2013). Before the experiment, animals were sedated with 3% isoflurane in medical air at a flow rate of 0.4 L/min. During the MRI experiment, anesthesia was maintained at 1–2% isoflurane through a customized anesthesia mask, and body temperature was maintained at 36–37°C with a heating pad. Rectal temperature, ECG signal and breathing rate were monitored throughout the measurements. All measurements were performed with respiratory gating and cardiac triggering.

Systolic function was measured from cine movies covering the whole left ventricle (15–18 frames/cardiac cycle) using prospectively cardiac‐triggered gradient echo imaging of 5–6 contiguous short‐axis and 2 long‐axis slices (slice thickness: 1 mm). The following imaging parameters were used for imaging: repetition time: 7 msec, echo time: 1.8 msec, flip angle: 15°, matrix: 192 × 192, and field of view: 30 × 30 mm^2^. For diastolic function, only the midventricular slice was acquired using retrospectively triggered gradient echo imaging, which allowed data reconstruction with a much higher temporal resolution (50–60 frames/cardiac cycle). The following imaging parameters were used for imaging: repetition time: 4.7 msec, echo time: 2.35 msec, flip angle: 15°, matrix: 128 × 128, field of view: 30 × 30 mm^2^, effective time resolution: 2 msec. Image segmentation and data analysis were performed using CAAS MRV 2.0 (Pie Medical, Maastricht, The Netherlands) or Segment (version 1.8 R1145, http://segment/heiberg.se).

### Measurement of arterial blood pressure

After induction of anesthesia, the left carotid artery was cannulated with a polyethylene tubing line with an outer diameter of 0.61 mm. For measurements of arterial blood pressure, the tubing line was connected to a transducer filled with phosphate‐buffered physiological saline solution containing 100 U/mL of heparin (Safedraw Transducer Blood Sampling Set, Argon Medical Devices, Texas, USA). Arterial blood pressure was continuously recorded using PowerLab software (PowerLab 8/35, Chart 7.0; ADInstruments Pty, Ltd., Castle Hill, Australia). Mean arterial blood pressure (MAP) was calculated after induction of anesthesia, but before MCE.

### Statistical analysis

Data are presented as mean ± SEM. The number of mice in individual experiments is shown in figure legends. Differences between groups were assessed by Mann–Whitney and time effects within groups were done by a two‐way ANOVA. Two‐tailed *P* values of less than 0.05 were considered statistically significant. Outliers were removed from data sets when samples were more than three times the interquartile range. Tests were performed using IBM^®^ SPSS^®^ Statistics version 22.0 (Chicago, USA).

## Results

### 5/6 nephrectomy surgery increases plasma FGF23 levels

**Figure 1:**
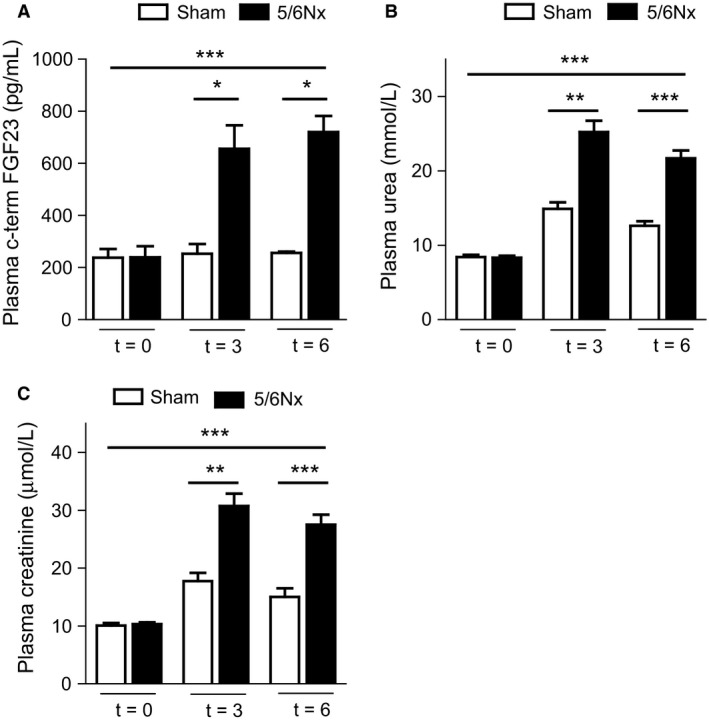
5/6Nx increases plasma FGF23 levels. (A) Sham surgery did not increase plasma FGF23 levels over time. 5/6Nx surgery increased plasma FGF23 levels 3 and 6 weeks after surgery by 2.7 and 2.8 fold, respectively, compared to sham mice. (B) 5/6Nx surgery increased plasma urea concentrations 3 and 6 weeks after surgery by 3.0 and 2.6 fold, respectively, which was significantly higher compared to sham mice. (C) 5/6Nx surgery increased plasma creatinine concentrations 3 and 6 weeks after surgery by 3.0 and 2.7 fold, respectively, which was significantly higher compared to sham mice. Data are mean ± SEM, *P ≤* *0.05 between groups performed by Mann–Whitney, ***P ≤* *0.001 for time effects performed by a two‐way ANOVA. For Figure 1A *n* = 3–4 for sham and *n* = 4–8 for 5/6Nx, for figures 1B and 1C *n* = 12 for both groups for *t* = 0 and *t* = 3 and *n* = 4 for sham and *n* = 7 for 5/6Nx for *t* = 2.

Plasma FGF23 concentrations three weeks after 5/6 nephrectomy surgery (5/6Nx) were higher compared to sham surgery (656 ± 37 vs. 253 ± 19 pg/mL, respectively, *P* = 0.011, Fig. 1A) and this differences sustained six weeks after surgery (720 ± 31 vs. 256 ± 3 pg/mL, *P* = 0.034). Sham surgery did not induce an increase in plasma FGF23 concentrations. There was a statistically significant interaction between the effects of time and surgery (e.g., 5/6Nx or sham) on FGF23 concentration (*P* < 0.001, Fig. 1A). Sham surgery did not induce an increase in plasma FGF23 concentrations. There was a statistically significant interaction between the effects of time and surgery (e.g., 5/6Nx or sham) on FGF23 concentration (*P* < 0.001, Fig. 1A). Plasma urea concentrations 3 weeks after 5/6 nephrectomy surgery (5/6Nx) were higher compared to sham surgery (25.2 ± 1.5 vs. 14.9 ± 0.9 mmol/L, respectively, *P* = 0.008, Fig. 1B) and these differences sustained six weeks after surgery (21.7 ± 1.0 vs. 12.6 ± 0.6 mmol/L, *P* < 0.001). There was a statistically significant interaction between the effects of time and surgery (e.g. 5/6Nx or sham) on plasma urea concentration (*P* < 0.001, Fig. 1B). Plasma creatinine concentrations 3 weeks after 5/6 nephrectomy surgery (5/6Nx) were higher compared to sham surgery (30.7 ± 2.2 vs. 17.8 ± 1.4 *μ*mol/L, respectively, *P* = 0.008, Fig. 1C) and these differences sustained 6 weeks after surgery (27.5 ± 1.7 vs. 15.0 ± 1.5 *μ*mol/L, *P* < 0.001). There was a statistically significant interaction between the effects of time and surgery (e.g., 5/6Nx or sham) on plasma creatinine concentration (*P* < 0.001, Fig. 1C).

### Calcium fluxes in cardiomyocytes are disturbed both after 5/6Nx and FGF23 injections

To assess whether 5/6Nx or FGF23 injections directly affect cardiomyocyte contractility and calcium handling, cardiomyocytes from 5/6Nx mice, FGF23 injected mice and control mice were isolated. After stimulation by 1 Hz to induce contraction, diastolic sarcomere length and fractional shortening in 5/6Nx and FGF23 injected mice were not different compared to control mice, and in addition, shortening and relaxation velocities were not changed after 5/6Nx or FGF23 injections (Table 1). Time to peak shortening was longer in 5/6Nx mice compared to sham (2.77 ± 0.19 vs. 2.41 ± 0.16 msec, respectively, *P* = 0.001), but was not different between PBS and FGF23 injected mice.

**Table 1:** 5/6Nx and FGF23 injections do not change shortening parameters

	Sham	5/6Nx	*P*‐value	PBS	FGF23	*P*‐value
Diastolic SL (*μ*m)	1.764 ± 0.004	1.761 ± 0.003	0.358	1.858 ± 0.009	1.851 ± 0.007	0.634
Systolic SL (*μ*m)	1.654 ± 0.006	1.640 ± 0.006	0.063	1.709 ± 0.011	1.702 ± 0.011	0.735
Sarcomere shortening (%)	6.23 ± 0.30	6.86 ± 0.30	0.148	7.98 ± 0.46	8.01 ± 0.54	0.967
Shortening velocity (*μ*m/sec)	−4.10 ± 0.20	−4.23 ± 0.18	0.499	−5.92 ± 0.39	−5.76 ± 0.39	0.793
Time to peak shortening (msec)	52.5 ± 0.9	56.6 ± 0.9	**0.001**	46.9 ± 1.9	48.7 ± 1.3	0.417
Relaxation velocity (*μ*m/sec)	2.41 ± 0.16	2.77 ± 0.19	0.367	4.64 ± 0.37	4.47 ± 0.34	0.847

Diastolic and systolic sarcomere length, sarcomere shortening (%), shortening velocity and relaxation velocity were not changed after 5/6Nx or FGF23 injections. Only time to peak shortening was increased after 5/6Nx, while this was not changed in FGF23 injected mice.

Data are mean ± SEM. *N* = 87–88 for sham, *n* = 99–100 for 5/6Nx, *n* = 34 for PBS, except for sarcomere shortening (%) where *n* = 30, and *n* = 29 for FGF23.

**Figure 2:**
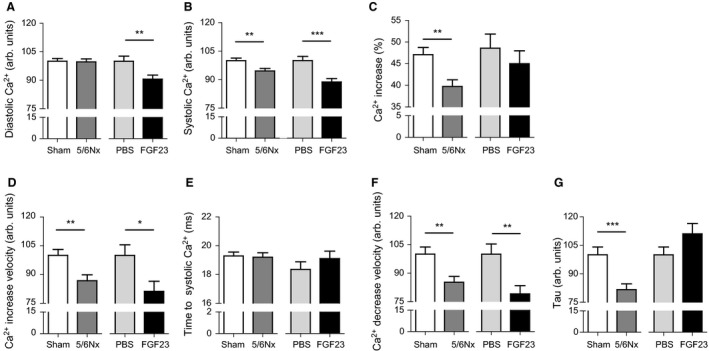
5/6Nx and FGF23 injections change cytosolic calcium content and calcium fluxes in cardiomyocytes. (A) Diastolic calcium content is decreased after FGF23 injections (−9.4%, *P* = 0.009) compared to control, but unchanged after 5/6Nx. (B) Cytosolic calcium content during systole is decreased after both 5/6Nx (−5.4%, *P* = 0.006) and FGF23 injections (−11.3%, *P* < 0.001) compared to control mice. (C) Relative calcium increase during systole is decreased in 5/6Nx mice compared to sham mice (39.7 ± 1.5 vs. 47.1 ± 1.7%, respectively, *P* = 0.001), but unchanged after FGF23 injections. (D) Calcium increase velocity during systole is decreased after 5/6Nx (−13.2%, *P* = 0.002) and after FGF23 injections (−18.7%, *P* = 0.025) compared to controls. (E) Time to peak calcium content during systole is unchanged after both 5/6Nx and FGF23 injections compared to controls. (F) Calcium decrease velocity during diastole is decreased in both 5/6Nx mice (−14.8%, *P* = 0.003) and in FGF23 injected mice (−20.9%, *P* = 0.005), compared to control mice. (G) Tau is decreased after 5/6Nx (−18.3%, *P* < 0.001) compared to control, but unchanged after FGF23 injections. Data are mean ± SEM. *N* = 87–88 for sham, *n* = 99–100 for 5/6Nx, *n* = 34 for PBS and *n* = 29 for FGF23. * *P *≤* *0.05, ** *P *≤* *0.01, *** *P *≤* *0.001. Statistical testing was done by Mann–Whitney for all figures.

Cytosolic calcium content during diastole was equal between sham and 5/6Nx mice, but lower in FGF23 mice compared to PBS mice (−9.4%, *P* = 0.009, Fig. 2A). Peak systolic calcium was lower in both 5/6Nx mice (−5.4%, *P* = 0.006, Fig. 2B) and FGF23 injected mice (−11.3%, *P* < 0.001) compared to control mice, and in addition, relative calcium increase during systole was lower in 5/6Nx mice compared to sham (39.7 ± 1.5 vs. 47.1 ± 1.7%, respectively, *P* = 0.001, Fig. 2C), but not different between FGF23 and PBS injected mice.

Maximal systolic calcium increase velocity was lower in both 5/6Nx mice (−13.2%, *P* = 0.002, Fig. 2D) and FGF23 injected mice (−18.7%, *P* = 0.025) compared to controls, although total time to peak systolic calcium was comparable between groups (Fig. 2E). Diastolic calcium decrease velocity was lower in both 5/6Nx mice (−14.8%, *P* = 0.003, Fig. 2F) and FGF23 injected mice (−20.9%, *P* = 0.005) compared to controls. Tau (time constant of relaxation) was only decreased in 5/6Nx mice (−18.3%, *P* < 0.001, Fig. 2G) and not in FGF23 mice.

### 5/6Nx and FGF23 injections do not change SERCA2a protein expression and phosphorylation status of PLN and cTnI

**Figure 3:**
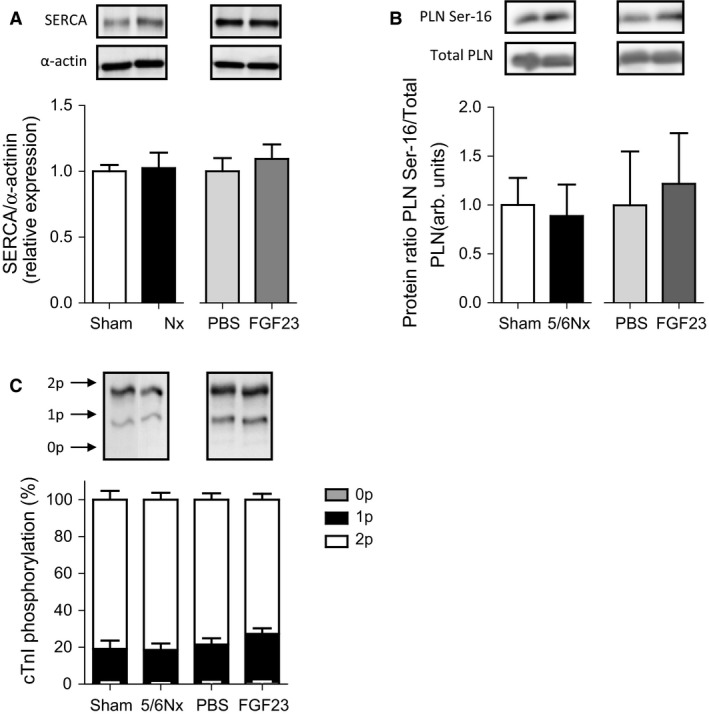
5/6Nx and FGF23 injections do not change SERCA protein expression, and PLN and cTnI phosphorylation. (A) No difference was observed after 5/6Nx surgery or FGF23 injections, compared to controls for SERCA protein expression. (B) Protein expression ratio of phosphorylated phospholamban (PLN Ser‐16) over total PLN was not changed after 5/6Nx or FGF23 injections compared to control groups. (C) Cardiac troponin I (cTnI) phosphorylation was determined by Phos tag analysis. No differences between groups were observed for bisphosphorylated (2p), monophosphorylated (1p) and unphosphorylated (0p) cTnI between groups. Data are mean ± SEM. *N* = 4–5 for all groups. Statistical testing was done by Mann–Whitney for all figures.

To explore the observed changes in calcium transients, we determined SERCA2a protein expression, the key calcium channel responsible for calcium reuptake in sarcoplasmic reticulum. No difference was observed after 5/6Nx surgery or FGF23 injections, compared to controls (Fig. 3A). Phosphorylation of PLN, the activator of SERCA2a, was also not changed between groups (Fig. 3B). Additionally, total PLN expression was not different between groups (data not shown).

Phosphorylation of cardiac troponin I (cTnI), the major determinant of myofilament calcium sensitivity of force development, was determined by Phos tag analysis. No differences between groups were observed for bisphosphorylated, monophosphorylated, and unphosphorylated cTnI between groups (Fig. 3C).

### 5/6Nx surgery and exogenous FGF23 concentrations do not induce left ventricular hypertrophy

**Figure 4:**
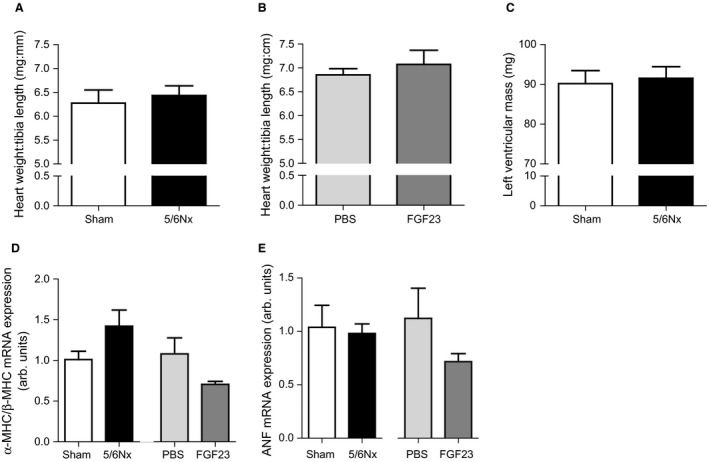
5/6Nx or FGF23 injections do not induce LVH and molecular markers of LVH. (A) Six weeks after sham or 5/6Nx surgery heart weight:tibia length was not different between groups. (B) One week after PBS or FGF23 i.p. injections no difference between groups was observed for heart weight:tibia length. (C) Left ventricular mass measured by MRI was not increased after 5/6Nx compared to sham. (D) mRNA expression of *α*‐myosin heavy chain (*α*‐MHC) over *β*‐myosin heavy chain (*β*‐MHC) ratio was not different between sham and 5/6Nx mice, or between PBS and FGF23 injected mice. (E) mRNA expression of atrial natriuretic peptide (ANF) was not different between sham and 5/6Nx mice, or between PBS and FGF23 injected mice. Data are mean ± SEM. Figure 5A‐C; *n* = 6–7 for sham and 5/6Nx, *n* = 15 for PBS and *n* = 13 for FGF23. Figure 5D+E; *n* = 3 for sham, *n* = 4–5 for 5/6Nx, *n* = 5 for PBS and *n* = 4 for FGF23. Statistical testing was done by Mann–Whitney for all figures.

Heart weight over tibia length ratio was not increased in 5/6Nx mice compared to sham mice (6.43 ± 0.20 vs. 6.28 ± 0.28 mg:mm, respectively, Fig. 4A) after 6 weeks. Also, FGF23 injections did not induce LVH compared to PBS injected mice (7.07 ± 0.30 vs. 6.86 ± 0.13 mg:mm respectively, Fig. 4B). In addition, left ventricular mass measured by MRI was not increased in 5/6Nx mice 6 weeks after surgery compared to sham mice (91.5 ± 2.9 vs. 90.2 ± 3.3 mg, respectively, Fig. 4C), nor was the observed end‐diastolic volume, as a marker of ventricular diastolic function, different between groups (Table 2).

**Table 2:** Cardiac function is unchanged after 5/6Nx surgery

	Sham	5/6Nx	*P*‐value
Cardiac output (L/min)	4.80 ± 0.39	4.76 ± 0.25	0.886
Ejection fraction (%)	73.3 ± 1.6	72.4 ± 0.9	0.886
Stroke volume (*μ*L)	43.9 ± 2.6	43.5 ± 1.9	1.000
End‐systolic volume (*μ*L)	16.4 ± 2.1	16.7 ± 1.1	0.886
End‐diastolic volume (*μ*L)	60.2 ± 4.6	60.2 ± 2.9	0.886
E/A ratio	1.88 ± 0.29	1.83 ± 0.18	1.000

MRI was used to assess cardiac function in mice. Systolic function parameters, that is, cardiac output, ejection fraction, stroke volume and end‐systolic volume were not different between sham and 5/6Nx mice. Also diastolic function parameters, that is, end‐diastolic volume and E/A ratio were not changed between groups.

Data are mean ± SEM. *N* = 6 for sham and *n* = 7 for 5/6Nx, except for E/A ratio where *n* = 5 for 5/6Nx.

To confirm the absence of structural changes compatible with LVH, mRNA expression of *α*‐myosin heavy chain (*α*‐MHC), *β*‐myosin heavy chain (*β*‐MHC), and atrial natriuretic peptide (ANF) were determined in cardiac tissue. *α*‐MHC/*β*‐MHC mRNA expression ratio was not altered after 5/6Nx, nor after FGF23 injections (Fig. 4D). Also both total *α*‐MHC mRNA and total *β*‐MHC mRNA expression were not different between groups (data not shown). Finally, ANF mRNA expression was not altered in cardiac tissue of 5/6Nx and FGF23 injected mice compared to control groups (Fig. 4E).

### 5/6Nx does not impair cardiac function in vivo

**Figure 5:**
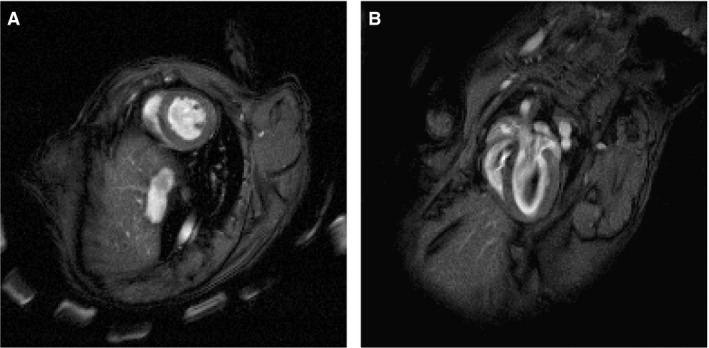
Cine cardiac MRI images of a mouse heart. (A) Short axis view of right ventricle and left ventricle. (B) Four chamber long axis view.

**Figure 6:**
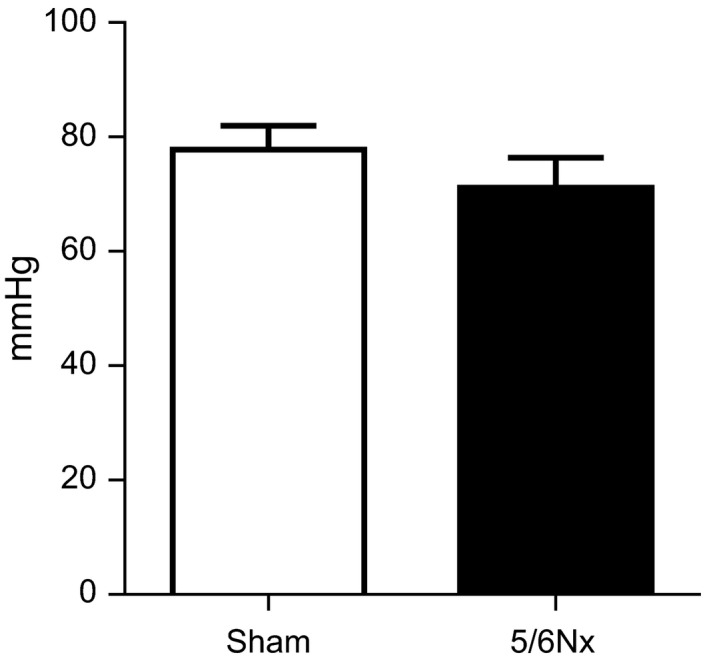
5/6Nx does not increase mean arterial blood pressure. Mean arterial blood pressure was not different between groups 6 weeks after sham or 5/6Nx surgery. *N* = 5 for sham and *n* = 6 for 5/6Nx. Statistical testing was done by Mann–Whitney.

To test whether kidney failure impairs cardiac function in vivo, we used magnetic resonance imaging (MRI) to sham and 5/6Nx mice (Fig. 5). Six weeks after 5/6Nx surgery, cardiac function was not different compared to sham mice (Table 2). Systolic function, measured as cardiac output, ejection fraction and stroke volume was not different between groups. Also diastolic function, measured as E/A ratio was not different between sham and 5/6Nx mice, and blood pressure was similar between the two groups (Fig. 6). Based on the absence of effect of 5/6Nx, the isolated effect of exogenous FGF23 was not assessed.

## Discussion

This study shows that CKD and increased FGF23 in isolation disturb kinetics of both systolic and diastolic calcium fluxes in cardiomyocytes, in the absence of changes in cardiomyocyte contractility and hypertrophy. These disturbances consist of decreased calcium influx and efflux velocities. In addition, CKD and FGF23 lower systolic calcium content in cardiomyocytes. Despite these changes in cardiomyocyte calcium handling, sarcomere length and contractility were unchanged, indicating an increased contraction propensity and early molecular markers of functional stiffness. We explored several potential underlying mechanisms including SERCA abundance, phosphorylation of PLN and cTnI, but none differed between groups. We postulate that either increased calcium sensitivity or an increased maximal force‐generating capacity may exist in CKD and FGF23. The calcium handling abnormalities were accompanied by an increase in plasma FGF23 in CKD, and could be mimicked in mice with normal kidney function by raising FGF23 concentrations. This suggests that the abnormalities in cardiomyocytes found in CKD could be ascribed at least in part to FGF23. The lack of change in sarcomere shortening and lengthening, despite abnormal calcium kinetics, was confirmed by real‐time in vivo measurements of parameters of cardiac function using MRI.

The lack of development of LVH and molecular markers of its development in our study, which seemingly contrasts with previous findings (Faul et al. 2011), can most likely be explained by the different setup of experiments. Faul et al. performed 5/6Nx in rats resulting in high blood pressure, while we used mice that were normotensive. In addition, the serum FGF23 concentration in rats was 13‐fold higher as compared to control, while we only observed 3‐fold increases in serum FGF23 concentrations. Faul et al. injected lower concentrations of recombinant FGF23 (80 vs. 160 *μ*g/kg/day) and duration of injections was 2 days shorter compared to our study, but recombinant FGF23 was injected intravenously in mice, while we injected intraperitoneally. Overall, our models most likely had insufficient exposure to FGF23 for LVH development. Due to this, however, we were able to establish the early phenomena of cardiomyocyte dysfunction.

The effect of experimental renal failure on cardiac function and cardiomyocyte calcium handling varies among studies. While in one study cardiac dysfunction in the absence of cardiomyocyte calcium disturbances was observed (Winterberg et al. 2016), others showed that cardiac dysfunction was accompanied by altered calcium channel expression (Kennedy et al. 2003; Hsueh et al. 2014). In contrast, we found calcium disturbances in the absence of cardiac dysfunction. The use of different species and genetic backgrounds might explain the differences between these studies. Also, disturbed calcium handling was observed in the presence of hypertension and LVH in these previous studies, and therefore it cannot be excluded that LVH itself, once established, contributed to disturbed calcium handling. However, in our models, LVH did not develop, possibly due to the short duration of the study, the relative low concentrations of FGF23 and mouse strain used. The absence of LVH or cardiac dysfunction indicates that disturbed calcium handling might be an early feature in cardiomyocytes, potentially preceding the development of LVH and cardiac dysfunction in CKD. Alternatively these abnormalities occur in parallel with more pronounced increases of FGF23, but then the independent subtle effects on calcium fluxes as we found here, cannot be distinguished from LVH.

We found that the velocity of calcium decay was reduced in both 5/6Nx mice and mice receiving FGF23 i.p. injections, which has been observed before in failing human hearts (Gwathmey et al. 1987; Beuckelmann et al. 1992; Piacentino et al. 2003). SERCA2a is the protein responsible for calcium reuptake into the sarcoplasmic reticulum (SR), and in both animal models of heart failure and human heart failure samples, SERCA2a protein expression was decreased (Hasenfuss 1998; Pieske et al. 1999; Bers 2006). Phosphorylation of PLN increases the activity of SERCA2a, and thereby increases calcium decay velocity (Simmerman and Jones 1998). In contrast, unphosphorylated PLN acts as an inhibitor on the SERCA2a pump. While levels of the PLN protein mostly remain unchanged in heart failure, the phosphorylation status of PLN is often decreased (Schwinger et al. 1999; MacLennan and Kranias 2003), and results in a decreased calcium uptake velocity by the SR.

In our model of renal failure, both SERCA2a protein expression and phosphorylation status of PLN were not changed, indicating that these cannot explain the declined velocity of calcium reuptake into the SR. This points to a more complex dysregulation. Since we did not observe quantitative changes in protein expression, alternatively calcium channel function might be affected by posttranslational modification in our models. The observed altered calcium fluxes with increased exogenous FGF23 concentrations may be caused by increased production of reactive oxygen species (ROS), as a recent study showed that FGF23 increases ROS formation in endothelial cells (Richter et al. 2016). In cardiomyocytes, ROS increase the open probability of RyRs, followed by its inactivation (Holmberg et al. 1991), and prolonged exposure of cardiomyocytes to O_2_^−.^ causes depletion of SR calcium as a result of PLN‐independent inhibition of SERCA (Zima and Blatter 2006). Also, S‐glutathionylation (protein modifications by oxidative stress) of troponin I increases calcium sensitivity (Breitkreuz and Hamdani 2015). FGF23‐induced ROS formation might therefore explain the observed changes in calcium fluxes in cardiomyocytes.

In contrast to our study, Touchberry et al. (2013) found that acute FGF23 treatment of cardiomyocytes increased intracellular calcium and improved contractility. However, in that study, the effect of increased FGF23 was examined ex vivo and thus showed acute effects of FGF23 on cardiomyocyte function in concentrations (18,000 pg/mL) that do not occur in vivo. Moreover, the phenotype of the model used, that is, increased contractility, does seem to correlate with clinical CKD only partially. In our study, we modeled physiological and chronic increases of FGF23 in vivo to optimally represent the clinical situation of patients with CKD. As FGF23 is chronically increased in patients with CKD, it is more likely that increased FGF23 concentrations do not improve cardiomyocyte contractility, but rather disturb calcium fluxes, possibly partly explaining early cardiac disease in CKD patients, especially diastolic heart failure, also in the absence of overt hypertrophy.

Our study has some limitations. We did not determine all calcium channel abundances and activities, for example, ryanodine receptor (RyR) expression or function. This calcium pump is mainly responsible for the cytosolic calcium increase during systole, but the decreased diastolic calcium content in our study indicates that there is no calcium leak from the SR into the cytosol by leaky RyRs, as often observed in heart failure (Yano et al. 2005). It is therefore unlikely that RyRs are functionally altered in our models. Another reason for the altered calcium decrease velocity in our models could be a change in NCX1 expression, another channel facilitating calcium reuptake by the SR, which we did not assess. However, a mouse study showed that only ~10% of calcium is removed by NCX and ~90% by SERCA, indicating that SERCA is mainly accountable for calcium removal from the cytosol in mice (Li et al. 1998). On the other hand, we cannot exclude that NCX has a more prominent role in our models. Moreover, this leaves the observation of attenuated calcium reuptake following 5/6Nx and exogenous FGF23.

Another limitation of our study is the lack of MRI data of FGF23 injected mice. We cannot rule out that these mice developed cardiac dysfunction, in contrast to 5/6Nx mice.

The strength of our study is that we used two mouse models in parallel; CKD mice, and non‐CKD mice with high exogenous FGF23 concentrations, both models with chronically increased circulating FGF23 levels, therefore representing chronic clinical exposure to FGF23 in a CKD setting or not. Also, we assessed the role of increased FGF23 concentrations in vivo on cardiomyocytes, while most studies increased FGF23 concentrations ex vivo. In addition, we used murine MRI to assess heart function in vivo and isolated cardiomyocytes from animals exposed to the same experimental conditions as in the in vivo experiments. Moreover, the concentrations of FGF23 we achieved may represent relative early clinical CKD, a situation comparable to our experimental conditions in terms of absence of abnormal mineral concentrations.

The prevalence of any cardiovascular disease is doubled in patients with CKD compared to those without CKD (U.S. Renal Data System, 2014) and death from cardiovascular causes is more common in these patients than progression to ESRD. Congestive heart failure (CHF) is the most common cardiovascular condition among CKD patients, reaching nearly 30% of patients above the age of 66 compared to 6% among patients of the same age‐group but without CKD (U.S. Renal Data System, 2014). Our findings strengthen the assumption that CKD‐induced increase in FGF23 may account for an important part to this risk.

FGF23 is highly increased in CKD patients and showed to contribute to abnormalities in cardiomyocyte structure and function (Faul et al. 2011; Touchberry et al. 2013). Here we demonstrate that this hormone directly affects cardiomyocyte function by disturbed calcium handling and is a key step in the pathogenesis of diastolic heart failure (Hamdani et al. 2008). This disturbed calcium handling induced by high FGF23 concentrations might therefore explain part of the increased incidence of heart failure and cardiac mortality in CKD patients. Future therapies should therefore focus on decreasing FGF23 concentrations or selectively block this effect of FGF23 on cardiomyocyte function in patients with CKD (Grabner et al. 2017) to reduce the risk of heart failure, even before the occurrence of LVH and cardiac dysfunction, and possibly also before the onset of overt hyperphosphatemia.

In conclusion, we have shown for the first time that slightly increased FGF23 concentrations in early stages of CKD induced disturbed calcium fluxes in cardiomyocytes that are independent from left ventricular hypertrophy and global cardiac dysfunction. Therefore, FGF23 might be a potential target to prevent cardiac disease in patients with CKD.

## Conflict of Interest

MGV received consulting and/or speaking fees from Medice, FMC, AbbVie, Baxter and Amgen, and grant support from Shire, Pfizer, Amgen and AbbVie. MH is a shareholder in Ionoptix ltd of which equipment was used to study calcium/contractility transients. ECE received grant support from the Netherlands Organization for Scientific Research (VIDI grant 917.133.72).
